# Host Use Patterns by the European Woodwasp, *Sirex noctilio*, in Its Native and Invaded Range

**DOI:** 10.1371/journal.pone.0090321

**Published:** 2014-03-27

**Authors:** Matthew P. Ayres, Rebeca Pena, Jeffrey A. Lombardo, Maria J. Lombardero

**Affiliations:** 1 Department of Biological Sciences, Dartmouth College, Hanover, New Hampshire, United States of America; 2 Departamento de Producción Vegetal, Universidad de Santiago, Lugo, Spain; CNRS, Université de Bourgogne, France

## Abstract

Accelerating introductions of forest insects challenge decision-makers who might or might not respond with surveillance programs, quarantines, eradication efforts, or biological control programs. Comparing ecological controls on indigenous vs. introduced populations could inform responses to new introductions. We studied the European woodwasp, *Sirex noctilio*, which is not a pest in its native forests, is a serious invasive pest in the southern hemisphere, and now has an uncertain future in North America after its introduction there. Indigenous populations of *S. noctilio* (in Galicia, Spain) resembled those in New York in that *S. noctilio* were largely restricted to suppressed trees that were also dying for other reasons, and still only some dying trees showed evidence of *S. noctilio*: 20–40% and 35–51% in Galicia and New York, respectively. In both areas, *P. sylvestris* (native to Europe) was the species most likely to have attacks in non-suppressed trees. *P. resinosa*, native to North America, does not appear dangerously susceptible to *S. noctilio*. *P. radiata*, which sustains high damage in the southern hemisphere, is apparently not innately susceptible because in Galicia it was less often used by native *S. noctilio* than either native pine (*P. pinaster* and *P. sylvestris*). Silvicultural practices in Galicia that maintain basal area at 25–40 m^2^/ha limit *S. noctilio* abundance. More than 25 species of other xylophagous insects feed on pine in Galicia, but co-occurrences with *S. noctilio* were infrequent, so strong interspecific competition seemed unlikely. Evidently, *S. noctilio* in northeastern North America will be more similar to indigenous populations in Europe, where it is not a pest, than to introduced populations in the southern hemisphere, where it is. However, *S. noctilio* populations could behave differently when they reach forests of the southeastern U.S., where tree species, soils, climate, ecology, management, and landscape configurations of pine stands are different.

## Introduction

International trade is leading to the introduction and establishment of many non-indigenous herbivores [1], [2]. Ecological invasions by plant pests are a globally important driver of undesirable changes in forest ecosystems [3] with broad socioeconomic ramifications [4]. The rate of invasions by plant pests is accelerating due to inexorable propagule pressure from expanding shipping [5]–[7]. Furthermore, the potentially vulnerable ports of entry are probably expanding poleward with climate warming [8], [9], with consequences for global trade [10].

Most introductions do not result in establishment of a population and most herbivores that become established are relatively benign (e.g., do not appear as important in forest health assessments) [11]–[13]. However, some newly established herbivore populations attain high abundance and become important new pests [14]. Thus it is a challenge for managers to develop appropriate responses following the detection of new non-indigenous species. Eradication efforts, quarantines, biological control and even detection systems [15], [16] can be expensive in terms of direct costs, investment of human expertise, and collateral damage (e.g., lost trade as a result of quarantines [17]). Thus there is a need for increased capacity to predict which species are likely or unlikely to become consequential pests if they become broadly established in a new region. An example is the recent detection of the European woodwasp, *Sirex noctilio* Fabricius in North America.

*S. noctilio* is not regarded as a pest within its native Europe [18], [19]; there, it is generally described as a relatively benign scavenger of dying pine trees and an element of native biodiversity in healthy forest ecosystems [20] (but see Wolf [21]). However, *S. noctilio* has repeatedly emerged as a very important pest of pines where it has been introduced in the Southern Hemisphere – first in New Zealand, since at least 1900, and since then in Australia in 1951, followed by Uruguay, Argentina, Brazil, South Africa, and Chile [22]–[32]. Populations of *S. noctilio* were discovered in North America in 2004, apparently following introduction into ports on Lake Ontario (e.g., Oswego in north-central New York State) within the St. Lawrence Seaway [33]. As was logical given experiences in the Southern Hemisphere, this discovery generated immediate attention and evaluation of possibilities for quarantines, eradication, and biological control [34]–[37]. This highlighted the question of whether *S. noctilio* in North America will display the pestilence that has been typical in the Southern Hemisphere or will be relatively benign as in their native European forests. If the latter, resources that might be invested in actively managing *S. noctilio* would perhaps be better directed towards preventing further invasions or managing those already established that more clearly pose threats to North American forests (e.g., emerald ash borer, Asian long-horned beetles, woolly adelgids, and gypsy moths; [38]–[42]). The possibility that *S. noctilio* is not destined to be a meaningful pest in North America is supported by the examples of European pine sawfly (*Neodiprion sertifer* (Geoffroy)) and pine shoot beetle (*Tomicus piniperda* (L.)), both of which can be damaging to pine in their native Europe, but which have only occasionally been noted as pests following their introductions (ca. 1925 and 1992, respectively) to the same North American pine forests as have now been colonized by *S. noctilio* [43]–[45].

We employed what could be a reasonably general tactic for evaluating the risks posed by a newly established non-indigenous herbivorous insect. We conducted studies in both the native and newly colonized ecosystems to compare the native and introduced insect populations with respect to their abundance, host use patterns, and aggressiveness (tendency to attack and kill trees that would otherwise survive). In the process, we aimed to better understand the factors that limit abundance of indigenous populations of *S. noctilio*. The premise is that understanding why a species is not a pest in one ecosystem can aid in predicting if those same controls are likely to operate in the newly invaded ecosystem. *S. noctilio* in Europe, like most populations of forest insects that are not pests, is poorly studied with respect to controls on abundance. So it is presently unknown why *S. noctilio* is not more abundant than it is in European forests. In the present study, we specifically evaluated the hypothesis that the abundance of European *S. noctilio* is limited by interspecific competition from other wood boring (xylophagous) insects. If supported, this would suggest an easy explanation for the outbreaks of *S. noctilio* in the Southern Hemisphere, where pine is not indigenous and where there is a correspondingly depauperate community of forest insects that can colonize pine trees [46].

Thus our research addressed the following questions. Is *S. noctilio* in North America likely to become a consequential pest as in the Southern Hemisphere or will it be no more aggressive in North America than in its native forests? How does the aggressiveness of *S. noctilio* compare among pine species: e.g., Scots pine, *Pinus sylvestris* L. (an ancestral host for *S. noctilio* that is native to Europe but which is also now widely planted and naturalized in North America) vs. red pine, *Pinus resinosa* Aiton (new North American host for *S. noctilio*)? Does interspecific competition for suitable host trees limit European populations of *S. noctilio*?

## Materials and Methods

### Ethics statement

Field studies in the U.S. were conducted on public land with the permission of Finger Lakes National Forest. Field studies in Galicia were mainly on land managed by the Galician Forest Administration (with the permission of Conselleria de Medio Rural, Xunta de Galicia) but also included some private land (with the permission of individual landowners). No protected species were sampled or harmed. The captures and handling of insects were in compliance with animal care policies in Spain and the U.S.

### Galicia, Spain

The silviculturally important landscapes of central Galicia (northwestern Spain) hold about 434,000 ha of pure pine stands [47], most of which are small (usually <1 ha), even-aged, and intensively managed by comparison to our study forests in New York State. The most common species is the native maritime pine (*Pinus pinaster* Aiton), which attains high productivity in this region (≈10 m^3^ ha^−1^ yr^−1^). However, since about 1970, there has been increased propagation of radiata pine (*Pinus radiata* D. Don), native to California, which can have even higher productivity than *P. pinaster* [48]–[50]. Also, there are some stands of native Scots pine (*P. sylvestris*).

#### Sirex detection

Most of the pine stands in Galicia are intensively managed. It was initially evident that *S. noctilio* were not present except in stands where there was a recent history of dying pines, most commonly because the stand had not been thinned and was experiencing self-thinning, but sometimes because of storm damage or other causes. By driving country roads, we identified 86 pine stands distributed over ≈20,000 km^2^ that contained some dead or dying pines. Then we surveyed each of these stands on foot for evidence of *S. noctilio*, (resin drips, emergence holes, and feeding galleries), which we found in 39 of these stands. In parallel, we deployed one baited Lindgren funnel trap in each of 17 localities distributed across the four provinces of Galicia (encompassing the regional spectrum of climatic conditions). Traps were baited with alpha-pinene (0.11 g/d elution, Pine Shoot Beetle lure, L1-3230/020, Contech Enterprises) and ethanol (0.01 g/d from our own elution devices). Traps were checked every two weeks from April 2009 to April 2010.

#### Quantifying infestation patterns

From the 39 stands in Galicia with evidence of siricids, we selected for more detailed sampling 6 stands of *P. pinaster*, 12 of *P. radiata*, and 5 of *P. sylvestris*. These 23 stands were selected to ensure that all three pine species were represented, and also with practical consideration of distance among plots, access to the stand, and availability of information about management history. Within each of the 23 stands, we examined each pine tree within a plot of 20×20 m centered on a tree with symptoms of *S. noctilio*. We measured the diameter at breast height (DBH) of each tree and scored each as alive, dying, or recently dead (judged to be within two years based on retention of needles and fine shoot tips). We also scored each tree for evidence of *S. noctilio* [51], [52], which could be resin drips from ovipositor stings and/or emergence holes typical of *S. noctilio* (i.e., multiple circular holes of variable size from 3–7 mm diameter). Here and in New York, when there were *Sirex*-like emergence holes but no diagnostic resin drips, we were usually able to expose one or more feeding galleries with a hatchet to verify that they had been siricids and not other woodborers with unusually circular emergence holes. If we could not reach the *Sirex*-like emergence holes for excavation, we required that at least one hole within the cluster be of a smaller diameter than the common cerambycids & buprestids. More information within “Species identity of Siricids”.

#### Interspecific competition

Within the 23 Galician study plots, all dying and recently dead trees were subjected to further inspection for other insects and pathogens. We searched for emergence holes of bark beetles and woodborers and any other external symptoms of insects or pathogens. We also looked for potential competitors by lifting the bark with a hatchet in two areas of 20×20 cm in areas of the tree bole being used by *S. noctilio*. Finally, we made a cut at the level of the root collar to detect root fungi and inspected the crown for other insects and pathogens.

#### Influence of silviculture

We conducted an additional study within a long-term thinning trial near Lugo (Begonte, 43.150° N, 7.751° W) that has been established and maintained by the Sustainable Forest Management Unit of the University of Santiago de Compostela [53], [54]. The trees (*Pinus radiata*), were 18 years old at the time of our sampling (2010–11). There were 12 replicate plots (30×20 m, 15×10 rows of trees), half of which were thinned in 2004 and half of which were left as controls. We examined each tree (800–1400 per plot) as before for resin drips or emergence holes from siricids. We also recorded two measurements per plot of basal area (English BAF 10x prism).

### Finger Lakes National Forest, New York State

We also conducted studies in central New York State, within Finger Lakes National Forest (FLNF; ≈66 km^2^, 42.47°N, 76.78°W). This forest was well suited to our studies because it was only about 115 km south of a presumed port of entry (Oswego, NY) and had clearly been infested with *S. noctilio* for at least several years by the time of our first visits in 2009. There was good spatially explicit information on forest types and stand ages available from the U.S.D.A. Forest Service. Furthermore, we knew that *S. noctilio* populations in this area, unlike most other infested areas in New York, had been spared from impacts of sanitation cutting and trap trees. For North America, this was a relatively old and relatively undisturbed population of *S. noctilio* that was interacting with stands of even-aged, similarly managed hard pines, one species indigenous (*P. resinosa*) to the area and the other indigenous to Europe (*P. sylvestris*) where it is a natural host of *S. noctilio*. A weakness of our study was that this forest of only 65 km^2^ provided our only data from North America. On the other hand, the known distribution of *S. noctilio* in North America was still quite restricted [37], [55] so options were limited for study forests that had been interacting with *S. noctilio* for at least a few years.

The Finger Lakes National Forest included 114 ha of *P. resinosa* (32 stands) and 52 ha of *P. sylvestris* (primarily in three stands). We conducted intensive sampling within each of the three stands of *P. sylvestris* (13 ha planted in 1940, 22 ha planted in 1943, and 3.6 ha planted in 1952). We conducted matched sampling within a sample of four *P. resinosa* stands that were of similar size and age (5–20 ha, planted in 1940s) and intermixed within the same 6×3 km as the stands of *P. sylvestris*. Trees were older in NY than in Galicia but the diameters were similar. Sampling was designed to match or augment that conducted in Galicia. One change (following from our experience in Galicia) was that we used transects rather than plots to sample tree diameters and *Sirex* occurrence within stands. We sampled each stand with two, randomly placed, 50-m transects. At 10-m intervals along the transect we located the third nearest tree [56], and recorded its diameter at breast height (DBH). At three points along the transect (10, 30, and 50 m) we measured basal area (English BAF 10x prism), and in two stands of each species we also measured the height of the nearest tree.

We sampled for *S. noctilio* within stands by examining the area extending 25 m on either side of each transect. We located every recently dead or dying pine tree, measured its DBH, and, as in Galicia, scored each tree for presence or absence of *S. noctilio* based on resin drips and siricid emergence holes. In New York, where the growing season is shorter than in Galicia, the decline and subsequent insect colonization of trees goes more slowly. Therefore, recently dead trees in New York were defined as those judged to have died within 3 years (vs. 2 years in Galicia; based on the same criteria of retention of needles and fine shoot tips). In New York, in addition to the Galician protocol, a pair of researchers using close-focus binoculars independently estimated the number of siricid emergence holes from the lower bole (to base of live crown) of each recently dead or dying tree. We recorded crown class (dominant, co-dominant, intermediate, or suppressed), and assessed crown condition (topped, snapped, needles presence/absence and color). Finally, we examined neighboring live trees for evidence of *S. noctilio*.

### Comparisons of host use patterns among continents and pine species

All of our study stands in Galicia and New York were even-aged and had been planted evenly spaced. Thus the relative diameter of trees within a stand was strongly related to the status of individual trees within the crown. For each pine species in each study region, we calculated the diameter of individual trees relative to the average diameter within the stand (*DBH_is_*/*DBH_•s_*, where *DBH_is_*  =  diameter at breast height of each tree *i* within stand *s* and *DBH_•s_*  =  the average for the stand. This allowed us to compare the frequency distributions of *S. noctilio* host use relative to the size distributions of the trees with which they were competing, for each pine species in each region.

### Species identity of siricids

Our methods for diagnosing the presence of *S. noctilio* (generally following Hall [57], Dodds et al. [52], and Ryan et al. [51]) permitted the examination of many trees over a considerable area but also involved some uncertainty in species identification, which placed important limits on inferences from the studies. Resin drips from ovipositor stings are unique to *S. noctilio* (since it is the only siricid species attacking living trees; [20], [51], [52]). We observed resin drips from *Sirex noctilio* in all of our intensively measured study stands (23 and 7 stands in Galicia and New York respectively). However, resin drips were not evident in every infested tree. We found numerous individual trees within our measurement stands that had recently died (judged to be within two years), and which lacked visible resin drips but which contained clusters of emergence holes that appeared to be from *S. noctilio* (clusters of perfectly round holes 3–7 mm in diameter; feeding galleries with *Sirex*-like frass). Thus we employed “*Sirex*-like emergence holes” in our diagnostics, but interpreted them as upper estimates of the number of emerging *S. noctilio*. They were regarded as upper estimates because some *Sirex*-like emergence holes must have been from the parasitoids of *S. noctilio*, which leave similarly sized circular holes, and in NY, some *Sirex*-like emergence holes must have been from native species of siricids. Native siricids have been reported as rare relative to *S. noctilio* in our NY study area: emergences of siricids from infested pines were 95 to 99% *S. noctilio* in New York and Ontario [58]–[60], with the others being mainly *S. nigricornis* Fabricius. It seems reasonable that the siricid emergence holes we recorded in freshly dead trees in the Finger Lakes National Forest were also mainly *S. noctilio*. However, the native species of *Sirex*, who are presumably attracted to oviposit in freshly dead trees, might make up a greater proportion of exit holes in trees that had been dead for two years. We were conservative in scoring trees as positive for *S. noctilio* based on only *Sirex*-like emergence holes, but we could not reject the possibility of some false positives. As it turned out, this did not compromise our central result that vigorous trees in both study areas were very rarely infested by *S. noctilio*; *Sirex noctilio* cannot emerge from a tree without leaving *Sirex*-like emergence holes, even though there are other possible sources for *Sirex*-like emergence holes.

There was less uncertainty regarding *Sirex*-like emergence holes in Galicia. Spradbery and Kirk [20] found *S. noctilio* to be the only siricid in pines on the Iberian Peninsula and it is the only siricid that we have seen in the region. Other siricids reported from conifers on the Iberian Peninsula are *S. juvencus* L., *Xeris spectrum* (L.), *Urocerus gigas* (L.), *U. phantoma* (Fabricius) and *U. augur* (Mocsary) but these are evidently rare and isolated [61], [62] and typically feed in spruce or fir rather than pines [18], [20] but see [57], [61].

## Results

### Prevalence, aggression, and host use by *S. noctilio* in Galicia vs. New York State

**Figure 1:**
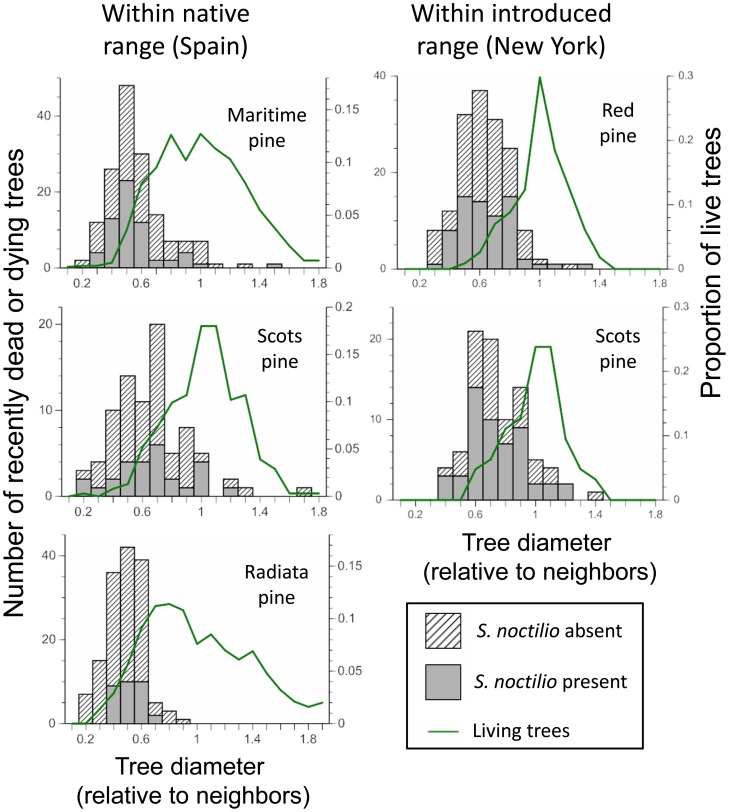
Insect infestations patterns. Comparison of infestation patterns by *Sirex noctilio* within their native range (Galicia, Spain) and their recently invaded range in New York State (Finger Lakes National Forest). Histograms indicate the size distribution of recently dead trees (with and without evidence of use by *S. noctilio*) relative to other trees within the even-aged stands (1.0 =  average dbh for the stand). Lines indicate the frequency distribution of living trees.

In Galicia, *S. noctilio* was detected in 39 of 86 pine stands that were pre-selected to be good habitat for *S. noctilio*. These stands were all in poor condition from the perspective of a forester. Within the 23 where we did a more detailed survey, about 10–50% of the standing trees were recently dead or dying and 3–71% of those had resin drips and/or emergence holes from *S. noctilio*: average of 40, 20, and 33% in stands of *P. radiata, P. pinaster*, and *P. sylvestris*, respectively (Table 1). In New York, the stands of *P. resinosa* were severely overstocked by forestry standards (basal area of 40 to 54 m^2^/ha) and 5–17% of the trees were recently dead or dying, 41–56% of which showed evidence of siricids from resin drips and/or emergence holes (Table 2). The *P. sylvestris* stands in New York were in very poor condition; high frequencies of trees that had previously died and fallen accounted for the low basal area (<50% that of *P. resinosa* stands that were of similar age and had been planted at the same densities on the same soils). Of the recently dead and dying *P. sylvestris* in New York, 20–69% had resin drips and/or emergence holes from siricids. In both Galicia and New York, dead and dying *P. sylvestris* were less likely than trees of the other pine species to show resin drips from stings by *S. noctilio* but tended to have more siricid emergence holes (Tables 1–2). In both Galicia and New York, *S. noctilio* were largely restricted to suppressed trees (smaller than average diameter) of the same size classes that were also dying for other reasons (Figure 1). In both areas, symptoms of *S. noctilio* were rare in non-suppressed trees (relative diameter ≥1) and were most frequent in Scots pine (native to Europe). In Galicia, our surveys only found 7 such trees, 5 of which were Scots pine, and in New York we only found 9 non-suppressed trees with resin drips from resin drips from *S. noctilio* stings and/or emergence holes from siricids, 6 of which were Scots pine. In both areas, there were many recently dead trees that showed no evidence of occupation by *S. noctilio* (resin drips or siricid emergence holes), suggesting that neither population was being limited by suitable host trees. Even in the most densely colonized stands that we surveyed (Scots pine in New York), >30% of the recently dead trees had escaped any detectable colonization by *S. noctilio* (Table 2).

**Table 1:** Study sites with *S. noctilio* in Galicia, Spain.

Pine stands in Galicia, Spain			Tree diam. (cm ± SD)	Percentage of trees dead or dying	Subsets of dead or dying trees		
Species	Stand	Basal area (m^2^/ha ± SD)			% with resin drips from *S. noctilio*	% with siricid emergences	% with siricids^a^
*Pinus pinaster*	Corgo - C1	45±1	21±5	10	33	5	38
*Pinus pinaster*	Punxín - P1	59	13±3	3	57	14	71
*Pinus pinaster*	Punxín - P12	66	14±4	11	4	4	8
*Pinus pinaster*	Punxín - P8	64	13±4	11	30	19	48
*Pinus pinaster*	Rabade - RA1	52±1	13±6	53	26	6	32
*Pinus pinaster*	Punxín - P9	52	13±4	8	40	0	40
*Pinus radiata*	Begonte - B2	49±1	17±7	20	0	3	3
*Pinus radiata*	Begonte - B3	47±1	16±7	18	4	4	7
*Pinus radiata*	Begonte - B5	40±1	17±6	24	5	8	14
*Pinus radiata*	Begonte - BZ10	51±1	18±7	21	13	3	17
*Pinus radiata*	Begonte - BZ12	45±1	17±7	12	11	0	11
*Pinus radiata*	Begonte - BZ2	48±1	17±5	25	11	5	16
*Pinus radiata*	Begonte - B1	30±1	20±6	4	67	0	67
*Pinus radiata*	Begonte - B11	26±1	20±5	11	10	10	20
*Pinus radiata*	Begonte - BZ1	28±1	22±6	3	50	0	50
*Pinus radiata*	Begonte - BZ3	36±1	19±7	17	0	6	6
*Pinus radiata*	Begonte - BZ4	36±3	20±7	23	12	4	16
*Pinus sylvestris*	Manzaneda - M1	52±3	24±5	25	0	58	58
*Pinus sylvestris*	Rodeiro - R1	30±1	18±4	11	6	11	17
*Pinus sylvestris*	Ancares - A1	57±1	27±4	34	0	18	18
*Pinus sylvestris*	Manzaneda - M2	43±3	28±5	10	0	50	50
*Pinus sylvestris*	Rodeiro - R2	63±4	16±4	7	0	14	14
*Pinus sylvestris*	Rodeiro - R3		22±4	47	4	38	42
*Pinus pinaster* (means ± SE)		56±3	15±1	16±7	32±7	8±3	40±8
*Pinus radiata* (means ± SE)		40±3	19±1	16±2	17±6	4±1	20±6
*Pinus sylvestris* (means ± SE)		49±6	23±2	22±7	2±1	32±8	33±8

a Resin drips from ovipositor stings by *S. noctilio* and/or emergence holes from siricids.

**Table 2:** Study sites with *S. noctilio* in Finger Lakes National Forest, New York.

Pine stands in Finger Lakes NF, New York				Percent of trees dead or dying	Subsets of dead or dying trees			
Pine species	Density (trees/ha)	Basal area (m^2^/ha±SD)	Tree diam.^a^ (cm±SD)		% with resin drips from *S. noctilio*	% with siricid emergences	% with siricids^b^	Siricid emergence holes per tree^c^ (± SD)
*P. resinosa*	469	40±5	30±3	9	27	32	50	11±9
*P. resinosa*	948	54±9	23±5	17	25	21	41	26±83
*P. resinosa*	1560	52±11	21±3	5	11	32	41	10±7
*P. resinosa*	1258	52±7	24±5	5	6	56	56	11±15
*P. sylvestris*	439	25±5	26±5	22	4	67	69	26±43
*P. sylvestris*	495	21±5	22±2	37	0	65	65	159±281
*P. sylvestris*	311	19±1	25±5	19	0	20	20	3±2
*P. resinosa* (means±SE)		46±6	24±2	9±3	17±5	35±7	46±3	15±3
*P. sylvestris* (means±SE)		20±3	24±1	26±5	1±1	50±15	51±15	63±48

a Tree heights (± SD): 1^st^ two *P. resinosa* stands  =  23.1±3.1 and 18.1±3.5 m; 1^st^ two *P. sylvestris* stands  =  20.1±4.7 and 18.0±1.8 m.

b Resin drips from ovipositor stings by *S. noctilio* and/or emergence holes from siricids.

c Only including trees from which there were some siricid emergence holes.

Insect trapping in Galicia (17 traps for one year) captured 2089 bark beetles (Scolytinae), 200 *Pissodes castaneus* De Geer, 56 Cerambycidae, 5 Buprestidae, and 62,000 other invertebrates representing 198 families in 27 orders. The same traps only caught two *S. noctilio* (both female). No other siricids were captured.

**Figure 2:**
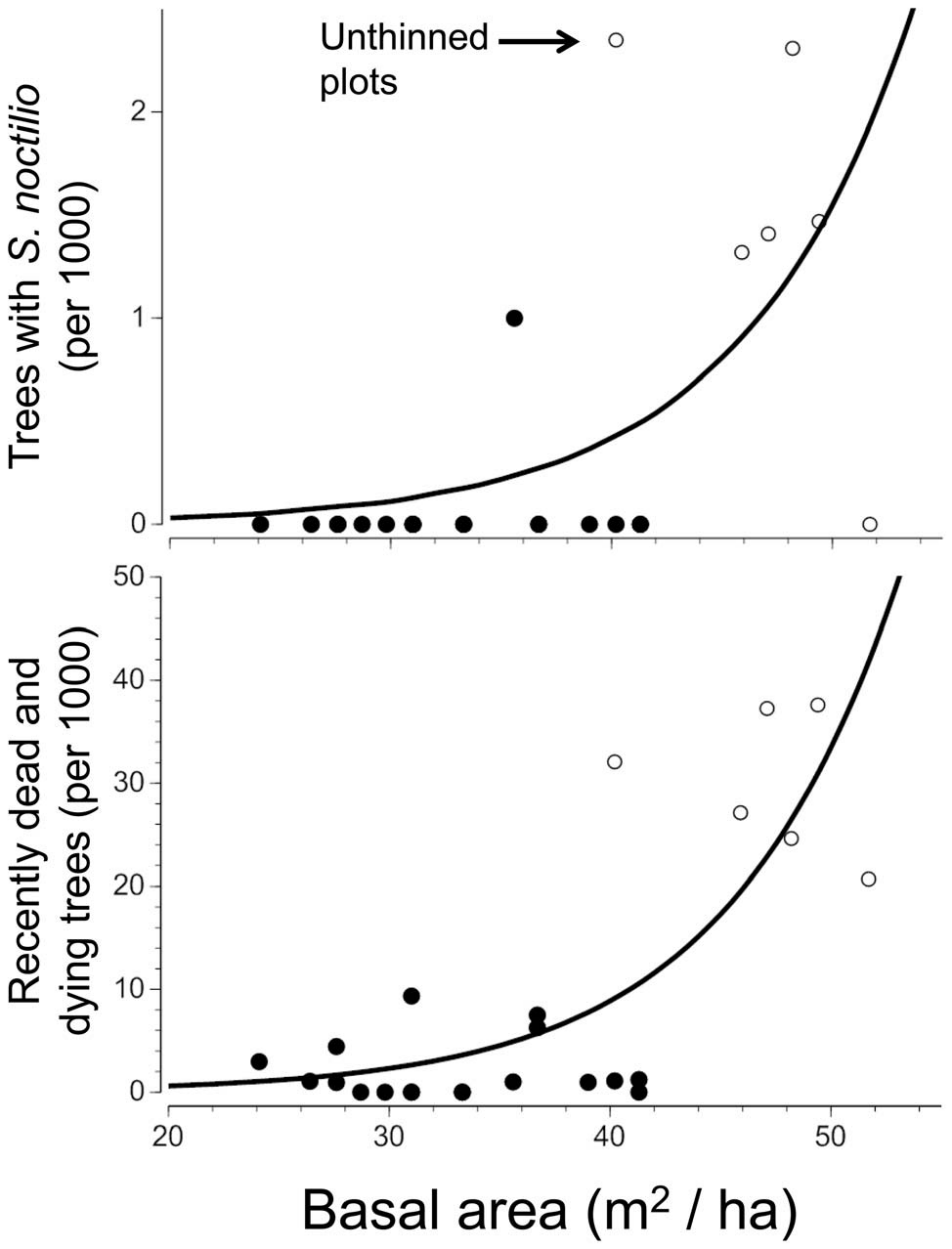
Infestations vs. stand density. Incidence of stings or emergence holes from *Sirex noctilio* (upper) and frequencies of dead and dying trees (lower) in experimental plots (thinned or unthinned) of *Pinus radiata* near Begonte, Galicia. Each point is one 20×30 m plot with 800–1400 trees. Trees were censused in summer 2011. Both logistic regressions were significant at *p*<0.001.

Censuses of thinned and unthinned plots of *P. radiata* at Begonte, Galicia reinforced the result that *S. noctilio* were mainly restricted to suppressed trees in overstocked stands (Figure 2): 5 of 6 unthinned stands (with basal area >40 m^2^/ha) contained trees with symptoms of *S. noctilio* while only 1 of 18 thinned plots had even a single tree with symptoms of *S. noctilio*. As expected, most of the dead and dying trees in general were in the unthinned, high basal area, stands, and most of these were dying without the involvement of *S. noctilio*: in the unthinned stands about 15-times more trees were dying from self-thinning than had resin drips or emergence holes from *S. noctilio* (Figure 2).

### Interspecific competition as a limit on *S. noctilio* abundance in Galicia?

Compilations from the literature supported the expectation that pine forests of western Europe harbor a diverse community of woodboring insects. Records indicated 20 species of Cerambycidae and 11 species of Buprestidae that feed in pines in Galicia, Spain; 7 and 4 of these species, respectively, can be described as common (Table 3). The region of eastern North America invaded by *S. noctilio* is even more diverse with respect to woodborer diversity: published records indicated 29 species of Cerambycidae and 9 species of Buprestidae, 7 and 1 of which could be described as common (Table 4). Dodds et al. [63] found 20 species of Cerambycids associated with *Sirex* trap trees near Syracuse, NY. In comparison to Europe and North America, pine forests of the Southern Hemisphere are dramatically depauperate. We could only find records of 8 species of Cerambycidae and 1 Buprestidae that feed on pines in the Southern Hemisphere – with no more than 2–4 in any region, and only one that has been described as common (Table 5).

**Table 3:** Wood boring beetles (cerambycids and buprestids) in pines^a^ of Galicia, Spain. After Vives ([62]) and Verdugo ([112]).

Cerambycdae	Common
*Ergates faber* (Linnaeus, 1758)	X
*Prionus coriarius* (Linnaeus, 1758)	X
*Nothorina punctata* (Fabricius, 1798)	
*Arhopalus ferus* (Mulsant, 1839)	
*Arhopalus rusticus* (Linnaeus, 1758)	
*Arhopalus syriacus* (Reitter, 1895)	
*Alocerus meosiacus* (Frivaldsky, 1838)	
*Spondylis buprestoides* (Linnaeus, 1758)	X
*Hylotrupes bajulus* (Linnaeus, 1758)	
*Rhagium (Rhagium) inquisitor* (Linnaeus 1758)	X
*Rhagium (Megarhagium) sycophanta* (Schrank, 1781)	X
*Rhagium (Megarhagium) mordax* (DeGeer, 1775)	X
*Rhagium (Hagrium) bifasciatum* Fabricius, 1775	X
*Aredolpona rubra* (Linnaeus, 1758)	
*Criboleptura strangulata* (Germar, 1824)	
*Pachytodes cerambyciformis* (Schrank, 1781)	
*Nustera distigma* (Charpentier, 1825)	
*Monochamus galloprovincialis* (Olivier, 1795)	
*Acanthocinus aedilis* (Linnaeus, 1758)	
*Pogonocherus (Pogonocherus) perroudi* Mulsant, 1839	
Buprestidae	
*Acmaeodera (Palaeotethya) bipunctata* (Oliver, 1790)	
*Chalcophora mariana* (Linnaeus, 1758)	X
*Buprestis (Buprestis) novemmaculata* (Linnaeus 1758)	
*Anthaxia (Haplanthaxia) paralella* Gory & Laporte, 1839	X
*Anthaxia (Haplanthaxia) scutelaris* Gene, 1839	
*Anthaxia (Melanthaxia) carmen* Obenberger, 1912	
*Anthaxia (Melanthaxia) nigritula* Ratzeburg, 1837	
*Anthaxia (Melanthaxia) sepulchralis* (Fabricius, 1801)	
*Melanophila cuspidata* (Klug, 1829)	X
*Phaenops cynaea* (Fabricius, 1775)	X
*Chrysobothris solieri* Laporte & Gory, 1836	

a Native pine spp  = *P. pinaster* and *P. sylvestris*; introduced  = *P. radiata*; all hard pines.

**Table 4:** Wood boring beetles (cerambycids and buprestids) of hard pines^a^ of northeastern North America ([113]–[115]. Asterisks indicate species reported by Dodds et al. ([63]), from New York in association with *Sirex noctilio* trap trees.

Cerambicidae	Common
Acanthocinus obsoletus* (Olivier, 1795)	
Acanthocinus pusillus* (Kirby in Richardson, 1837)	X
*Acmaeops discoideus* (Haldeman, 1847)	
*Acmaeops proteus* (Kirby, 1837)	
Aegomorphus modestus* (Gyllenhal in Schoenherr, 1817)	
Analeptura lineola* (Say 1824)	
Asemum striatum* (Linnaeus, 1758)	X
Astylopsis sexguttata* (Say, 1826)	X
Bellamira scalaris* (Say, 1827)	
Brachyleptura vagans* (Olivier, 1795)	
Brachyleptura champlaini* Casey, 1913	
*Callidium antennatum* (Newman, 1838)	
*Callidium schotti* (Schaeffer, 1917)	
Clytus marginicollis* (Laporte and Gory, 1835)	
Eupogonius tomentosus* (Haldeman, 1847)	
Evodinus monticola* (Randall, 1838)	
Microgoes oculatus* (LeConte 1862)	
Monochamus carolinensis* (Olivier, 1792)	X
Monochamus notatus* (Drury, 1773)	
Monochamus scutellatus* (Say, 1824)	X
*Monochamus titillator* (Fabricius, 1775)	
*Phymatodes dimidiatus* (Kirby in Richardson, 1837)	
Pogonocherus mixtus* (Haldeman, 1847)	
*Pygoleptura nigrella* (Say, 1826)	
Rhagium inquisitor* (Linnaeus, 1758)	
Stictoleptura canadensis* (Olivier, 1795)	X
Tetropium* cinnamopterum (Kirby in Richardson, 1837)	X
*Tetropium schwarzianum* (Casey, 1891)	
Xylotrechus sagittatus* (Germar, 1821)	
Buprestidae	
*Chalcophora virginiensis* (Drury, 1770)	X
*Chalcophora liberta* (Germar, 1824)	
*Chrysobothris dentipes* (Germar, 1824)	
*Chrysobothis harrisi* (Hentz, 1827)	
*Dicera* spp. (Eschscholtz, 1829)	
*Buprestis striata* (Fabricius, 1775)	
*Buprestis lineata* (Fabricius, 1775)	
*Melanophila acuminata* (De Geer, 1774)	
*Anthaxia inornata* (Randall, 1838)	

a Reported as feeding in one or more of *P. banksiana*, *P. resinosa*, and *P. virginiana*, all hard pines native to the region of eastern North America recently invaded by *S. noctilio*.

**Table:** **Table 5.** Wood borers (Cerambycidae or Buprestidae) of pines in the Southern Hemisphere ^a^.

	Countries in Southern Hemisphere where established						Region of origin	
Taxon	Argentina	Brazil	Chile	S.Africa	Australia	NZ		Reference
Coleoptera: Cerambycidae								
*Arhopalus syriacus* (Reitter, 1895)				X*			Europe	[115]
*Acanthinodero cummingi* a (Hope, 1833)			X				S. America	[116]
*Colobura alboplagiata* (Blanchard, 1851)	X		X				S. America	[116], [117]
*Delocheilus prionoides* Thomsen, 1860				X			S. Africa	[115]
*Diotimana undulata* (Pascoe, 1859)					X		Australia	[118], [119]
*Eriphus laetus* b (Blanchard, 1851)	X		X				S. America	[116]
*Hexatricha pulverulenta* (Westwood, 1843)						X	New Zealand	[120], [121]
*Prionoplus reituclaris* White, 1843						X	New Zealand	[120]
Coleoptera: Buprestidae								
*Buprestis novemmaculata* L.			X				Europe	[122]

*Sometimes common

a =  *Ancistrotus cummingii*

b =  *Callideriphus laetus*

During our surveys in Galicia, we discovered and inspected a total of 503 pine trees that were dying or had recently died. Overall, there were 120 of these that had resin drips or emergence holes from *S. noctilio* and there were frequent occurrences of six other classes of insects and pathogens that were candidates for interactions with *S. noctilio*: the sometimes aggressive bark beetle,*Tomicus piniperda*; other bark beetles within the Scolytinae; long-horned beetles (Cerambycidae); the weevil, *Pissodes castaneus*; metallic woodboring beetles (Buprestidae); and Armillaria fungus (Table 6). This community displayed a cluster of strong positive associations, with Scolytinae, Cerambycidae, *P. castaneus*, Buprestidae, and Armillaria all tending to co-occur in the same trees (commonly at 3- to 7-fold more frequent co-occurrence than expected by chance). By comparison, *S. noctilio* displayed only weak associations with the other species that feed in the same habitat. There was no evidence that other taxa avoided *S. noctilio*, or vice versa (no significant negative associations), and the positive associations were weak and limited to only *T. piniperda* and Cerambycidae (Table 7).

**Table 6:** Number and percentage of 503 dying or recently dead trees in Galicia, Spain that were infested by each of seven classes of insects or pathogens.

Insect or pathogen	N	%
*Sirex noctilio*	120	24
*Tomicus piniperda*	25	5
Scolytinae	100	20
Cerambycidae	43	9
*Pissodes castaneus*	11	2
Buprestidae	47	9
*Armillaria ostayea*	21	4
None of above	286	57

**Table 7:** Patterns of association^a^ among seven classes of insects and pathogens within 503 dying or recently dead pine trees in Galicia, Spain (Table 6).

	*Tomicus*	Scolyt	Ceram	*Pissodes*	Buprest	*Armillaria*
*Sirex noctilio*	2.0**	1.1	1.7*	0.4	1.3	1.0
*Tomicus*		2.8***	1.4	0	1.7*	1.0
Scolytinae			3.4***	1.9*	3.6***	3.6***
Cerambycidae				7.4***	7.0***	5.0***
*Pissodes*					5.8***	6.5***
Buprestidae						4.1**

a Entries are an index of association for each pair of organisms (ratio of observed over expected co-occurrences under null model of no association). Asterisks indicate significant non-random associations via Fisher exact test. Indices >1 indicate a positive association (tendency to co-occur in the same tree more than often than expected by chance).

## Discussion

### Prevalence and aggression

In both Galicia and New York, the majority of *S. noctilio* were in suppressed trees that were likely to die from competition in the next year or two with or without insect attacks (Figs. 1–2). We found only a few apparently vigorous trees that were being stung and potentially killed by *S. noctilio* (7 in Galicia, 9 in New York). This matches other observations in New York [52] and Europe [19], [20], [57] (but see [21]). Thus the aggressiveness of *S. noctilio* in the Finger Lakes region of New York State appears to be about as low as in its native Europe. For some forest insects, aggressiveness can increase with increases in local abundance, for example due to increased efficacy in overwhelming tree defenses [64], [65]. If *S. noctilio* follows this pattern, as has been reported from some outbreak situations in the Southern Hemisphere [32], [66] we would have expected a higher incidence of vigorous trees being attacked in New York vs. Galicia because the local abundance of *S. noctilio* was clearly higher in New York where there were frequently tens to hundreds of siricid emergence holes per infested tree (Table 2) whereas the number of holes in Galicia was much lower. These are favorable results from the perspective of forest managers and shareholders in North America because it implies that *S. noctilio*, at least in the region we studied, will be only a minor primary source of tree mortality and perhaps no more of a pest than in Galicia.

The very limited spatial extent of our studies in North America presents an important caveat to inferences regarding future impacts. The distribution of *S. noctilio* in North America is presently extending southwards [67] toward the extensive and highly productive pine forests of the southeastern U.S. where the soils, climate, pine species, landscape configurations of host trees, and community of associated species is different. Current information is inadequate to know if the aggressiveness of *S. noctilio* will be as low in southern pine ecosystems as at present in the Finger Lakes National Forest in New York. It is encouraging that none of the previously established non-indigenous insect herbivores of pines have yet emerged as notable pests in the southeastern U.S. (including *Tomicus piniperda*, even though there was considerable concern about this possibility 15–20 years ago; [68], [69]). Nonetheless, like others (e.g., Dodds et al. [52]), we find it premature to reject the possibility of meaningful future impacts of *S. noctilio* in North America, e.g., from regional harvest shortages in the forest products industry due to elevated tree mortality [70], [71].

### Host use

Our measurements in New York were consistent with previous evidence that the native *Pinus resinosa* is at less risk from *S. noctilio* than the non-indigenous *P. sylvestris*. Both Dodds et al. [52] and Eager et al. [59] estimated that siricids were about twice as likely to be found in *P. sylvestris* as in *P. resinosa*. We also found higher occurrence of siricids in *P. sylvestris* than *P. resinosa*, more emergence holes per infested tree, and a greater tendency to attack non-suppressed trees (Figure 2). It seems to be common for forest insects to have different effects on tree species with which they have interacted more or less in recent evolutionary time, but it can go in both directions. American ash is at risk of being eliminated from North America by the introduced Asian emerald ash borer (*Agrilus planipennis* Fairmaire) [72]. However there are also cases of plants from other continents being less susceptible to insect herbivores than the plant species with which the herbivore has evolved [73]. For example, the sawfly *Neodiprion annulus* Schedl tended to oviposit in its natural American pines over *P. sylvestris* from Europe [74] and stands of native *P. pinaster* in Galicia experienced more colonization by more species of stem-boring insects than the non-indigenous *P. radiata* [54]. Attraction of *S. noctilio* to *P. sylvestris* is partly related to host volatiles [75], which is consistent with higher trap captures of adult *S. noctilio* in stands of *P. sylvestris* than *P. resinosa* [16]. *P. resinosa*, at least when they are dying, seem to be as suitable for *S. noctilio* larvae as *P. sylvestris* because trap trees of the two species produced comparable numbers of emerging *S. noctilio* adults [16]. So *P. resinosa*, even if it is used less than *P. sylvestris*, is still recognized as a host by *S. noctilio*, can support development of *S. noctilio*, and – at least when suppressed – can apparently be killed by *S. noctilio*. Fortunately, *P. resinosa* is not dramatically susceptible like American ash are to emerald ash borers or eastern American hemlock and fir are to woolly adelgids [13]. Our conclusions agree with Dodds et al. [35], [52] that effects of S. *noctilio* on *P. resinosa* will generally be minor if stands are managed to avoid intense inter-tree competition. Our study stands of *P. resinosa* in NY were a worst case scenario in this respect in being unmanaged and overstocked (basal area >50 m^2^/ha in 3 of 4 stands vs. recommendations of 20–44 m^2^/ha; [76]); they were still experiencing only modest mortality from any sources, including *S. noctilio*. In contrast, the stands of *P. sylvestris* were in terrible condition from the perspective of a forester, had already lost half of their basal area and were continuing to lose trees due to *S. noctilio* and other sources (including *Tomicus piniperda*, *Ips pini* (Say), *Dendroctonus valens* LeConte, *Pissodes nemorensis* Germar, Armillaria root rot, and gall rust – probably *Endocronartium harknessi* (J.P. Moore) Y. Hiratsuka. It remains to be seen how *S. noctilio* will interact with other species of pines in North America.

Our study is the first that we know of to quantify use by *S. noctilio* in its native range of *P. radiata* vs. a native host species (but see [57]). *P. radiata*, native to California, is among the most widely planted tree species in the world, especially in the Southern Hemisphere [77]. At least in the landscape we studied in Galicia, it was not highly susceptible to *S. noctilio*. Actually, it was less susceptible than either of the native species, *P. pinaster* and *P. sylvestris*. A simple explanation is that the profile of secondary chemicals in *P. radiata* is not as good a match as native pines with the host-seeking behavior of *S. noctilio* female adults. This fits the general pattern for stem-boring insects of pine in Galicia, which are almost exclusively native at this time [54]. This result also makes it improbable that the persistent tendency for *S. noctilio* to become a pest in the Southern Hemisphere is because the most widely planted tree species, *P. radiata*, is intrinsically susceptible to this insect.

### Interspecific competition

In the forests of Galicia there are >30 other species of similarly-sized woodboring insects of pine (Table 3), which made it plausible that native populations of *S. noctilio* are at least partly limited by interspecific competition among xylophagous insects. They would clearly be released from such competition in the Southern Hemisphere (Table 5). This would be a favorable scenario for North America, where the diversity of xylophagous insects in pines is comparable to Europe (Table 4; [63]). However, more detailed patterns of occupancy within host trees failed to support a strong role for interspecific competition in determining the abundance of *S. noctilio* in Galicia. Most *S. noctilio* larvae were feeding in the absence of potential competitors and there were many apparently suitable trees that were unoccupied by *S. noctilio* or their putative competitors (Tables 6–7). Even though they feed on different tissue, it could be that phloem-feeding insects (bark beetles, chiefly Scolytinae) are more consequential than woodborers as (indirect) competitors of *S. noctilio*. In North America, Ryan et al. [78] found reduced *S. noctilio* emergence from trees that also contained bark beetles and hypothesized that fungal associates of bark beetles might outcompete the fungal mutualist of *S. noctilio* (see also [79], [80]). In Galicia, *Tomicus piniperda* seems the most likely bark beetle to be a strong indirect antagonist of *S. noctilio*, but we know of no data beyond the evidence that they tend to co-occur somewhat more frequently than by chance (Table 7). There would be merit to further study, and especially experimental tests [78], of interactions among *S. noctilio*, bark beetles, and their associated fungi.

Most damaging to the hypothesis of limitation via interspecific competition was the result that in its native forests, the supply rate of dying pines in Galicia apparently exceeds the ability of *S. noctilio* and its associates to colonize them. Of 503 dying or recently dead trees that we inspected, 286 (57%) were unoccupied by *S. noctilio* or any of its potential competitors (Table 6). Of 120 trees in this survey where we found *S. noctilio*, 77 lacked any of the potentially competing species. Furthermore, censuses of the Begonte plots indicated that *S. noctilio* were only colonizing about 1 in 15 trees that were dying from being overgrown (Figure 2). In the unthinned Begonte plots and most of the other study forests in Galicia where we found *S. noctilio* (Table 1), it was evident that pine trees had been dying regularly (a few deaths per thousand trees per year) for many years because there were many dead pines, standing and fallen, representing a range of decay states. Thus these stands seemed to be ideal circumstances for the resident population of *S. noctilio* to expand to the limits of the resource supply rate – if it were going to. Thus we reject the hypothesis that interspecific competition with other xylophagous species is a primary control on the abundance of *S. noctilio* in Galicia. A caveat to this conclusion is the possibility that pine trees in Galicia die at the wrong time of year with respect to the flight time of *S. noctilio* (are too well defended during one flight season and are too long dead the following flight season). This will require more knowledge of the relative timing of tree deaths, maximal susceptibility to *S. noctilio*, and flight times of *S. noctilio*.

### Other limitations on *S. noctilio* abundance in Galicia

Host suitability should not have been limiting to *S. noctilio* populations in our study areas in Galicia. The dominant indigenous pine species in Galicia, *P. pinaster*, was the most frequent host of *S. noctilio* (accounting for 45% of >8000 emergences) in the extensive surveys of Europe, Turkey, and North Africa by Spradbery & Kirk ([20]; with the other important hosts being *P. halapensis*, *P. sylvestris*, and *P. brutia* at 17, 12, and 11%). We also doubt that the landscape abundance of pine stands was limiting to *S. noctilio* in Galicia because there are >4000 km^2^ of pine forest and the distance among stands is generally modest relative to the flight capabilities of *S. noctilio* [81], [82]. Meteorological conditions can be a constraint on forest insect populations (e.g., [83]–[85]), but this is unlikely as a general explanation for the low abundance of *S. noctilio* in Galicia because the species occurs throughout the Iberian Peninsula and into North Africa; CLIMEX models indicate that *S. noctilio* is as well suited to Galicia as to places where it can be a pest [86].

Forest management must contribute to the low abundance of *S. noctilio* in Galicia, and probably Europe in general, because silvicultural practices (especially thinning) keep the basal area of most pine stands within 25–40 m^2^/ha, which is below stand densities at which we found most *S. noctilio* in experimental plots (Figure 2). This is consistent with reports from the Southern Hemisphere such as that of Jackson [87] that “at least 80% of the mortality [from *S. noctilio*] is always comprised of trees from the intermediate or suppressed sections of the stand” (see also [[29], [88]–[90]). Because pine forests of the Southern Hemisphere were shielded by distance from insect pests of pine for many decades, the economics favored a silvicultural tradition of growing pines at higher stocking levels than would be customary in the northern hemisphere (e.g., [91], [92] vs. [76], [93], [94]). If *S. noctilio* interacts with *P. radiata* there as it does in Galicia (Figure 2), management of stands to keep basal areas below that where self-thinning is strong should tend to limit damage from *S. noctilio* in the Southern Hemisphere as in Europe (see also [35]). Still, silviculture by itself seems inadequate to explain the low abundance of *S. noctilio* in Galicia, because there are unmanaged, high basal area stands in Galicia (as the ones we studied) and even in those stands *S. noctilio* populations do not seem to approach the limits of the resource supply rate from trees dying from self-thinning.

If resource quality, resource quantity, and climate are inadequate to explain the generally low abundance of *S. noctilio* in its native European forests, this implies the importance of top-down controls from natural enemies. The frequent success of biological control programs in the Southern Hemisphere [32], [95], [96] shows that enemies can limit the abundance of *S. noctilio*. Wolf [97] provided the best information we know regarding the ecology of enemies of *S. noctilio* in its native European forests. North American forests share all the most prominent enemies. As the most important predators in Europe, Wolf identified woodpeckers and the parasitoid wasp *Ibalia leucospoides* (Hockenworth). We did not quantify woodpeckers In New York, but we noted Pileated Woodpeckers (*Dryocopus pileatus*) foraging intensely and apparently efficiently in the *Sirex*-infested sections of *Sirex*-infested trees. In addition to woodpeckers, the invaded pine forests of North America also contained *I. leucospoides* (Holarctic distribution) before *S. noctilio* arrived. Within eight years of the discovery of *S. noctilio* in North America, parasitism by *I. leucospoides* was already comparable to Europe: about 20% and as high as 50% [58]–[60], [98]–[99] versus 20–30% (range of 15–75%) from vicinity of Belgium [97] and 36% from Mediterranean material [20]. The other two groups of *S. noctilio* enemies noted by Wolf for Western Europe, the rhyssine wasps (parasitoids) and the parasitic nematode *Deladenus siricidicola* (Bedding) are also already conspicuous in North American populations of *S. noctilio* [59], [60], [78], [100]–[102]. The pre-existence (or perhaps rapid unaided colonization for *D. siricidicola*) of diverse enemies that can also prey upon related indigenous siricids adds to ways in which the invasion of *S. noctilio* into North America is similar to Europe and different from the Southern Hemisphere. The ecology of *S. noctilio* in North America also involves such complexities as cleptoparasitoids [102] and transfer of symbiotic fungi among *Sirex* spp. [103]–[105]. The consequences for future population dynamics of such rich community interactions, including shared enemies, intraguild predation, and symbioses is partly understood in theory [106]–[108] but will hinge on still unknown details of the biology (e.g., relative preferences of *I. leucospoides* for *S. noctilio* vs. other *Sirex* spp.).

### Conclusions

Comparisons of ecology in native vs. newly colonized ecosystems can be a useful tactic for informing responses to new introductions of potential pest species. For example, management responses in Europe to the pinewood nematode, *Bursaphelenchus xylophilus* (Steiner and Buhrer) [109] and in China to the turpentine beetle, *Dendroctonus valens* LeConte [110], might be aided by better knowledge of what controls abundance of the corresponding indigenous populations in North America – where these species have not been well studied for the same reasons that *S. noctilio* has not been well studied in Europe. As knowledge grows of how differences in ecology between home and away can change the impacts of a species in its new environment, it may also become possible to better predict impacts from potential new invasives and specifically mitigate risks from those species for which propagule pressure is high and invasion consequences could be great. Given the global nature of invasion biology, international projects are surprisingly rare [111].
